# Supplementary material for: The effect of hybridization on transposable element accumulation in an undomesticated fungal species
Source: eLife. 2020 Sep 21;9:e60474. doi: 10.7554/eLife.60474 (PMC7584455; doi:10.7554/eLife.60474)
Supplement: Transparent reporting form [file elife-60474-transrepform.docx]

***eLife’s* transparent reporting form**

We encourage authors to provide detailed information *within their submission* to facilitate the interpretation and replication of experiments. Authors can upload supporting documentation to indicate the use of appropriate reporting guidelines for health-related research (see EQUATOR Network), life science research (see the [BioSharing Information Resource](https://biosharing.org/)), or the [ARRIVE guidelines](http://www.plosbiology.org/article/info:doi/10.1371/journal.pbio.1000412) for reporting work involving animal research. Where applicable, authors should refer to any relevant reporting standards documents in this form.

If you have any questions, please consult our Journal Policies and/or contact us: [editorial@elifesciences.org](mailto:editorial@elifesciences.org).

**Sample-size estimation**

- You should state whether an appropriate sample size was computed when the study was being designed
- You should state the statistical method of sample size computation and any required assumptions
- If no explicit power analysis was used, you should describe how you decided what sample (replicate) size (number) to use

Please outline where this information can be found within the submission (e.g., sections or figure legends), or explain why this information doesn’t apply to your submission:

For the wild strains, sample size was only limited by the collection of sequenced sampled isolates that met our high-coverage criterion (>35x). For the MA lines, the number of lines evolved was maximized given the available people who could perform passages by manual streaking, and the number of lines sequences was maximized given the resources available for sequencing.

- You should report how often each experiment was performed
- You should include a definition of biological versus technical replication
- The data obtained should be provided and sufficient information should be provided to indicate the number of independent biological and/or technical replicates
- If you encountered any outliers, you should describe how these were handled
- Criteria for exclusion/inclusion of data should be clearly stated
- High-throughput sequence data should be uploaded before submission, with a private link for reviewers provided (these are available from both GEO and ArrayExpress)

Please outline where this information can be found within the submission (e.g., sections or figure legends), or explain why this information doesn’t apply to your submission:

The growth measurements for the determination of the optimal sodium arsenite concentration (Figure 5-Figure supplement 7 was performed with a single replicate per strain and sodium arsenite concentration. The growth measurements in 0.8 mM on the collection of 123 strains was designed with 5 replicates. Excluding replicates that failed due to technical reasons, statistical analyses were performed on the strains which had 3 to 5 replicates.

- Statistical analysis methods should be described and justified
- Raw data should be presented in figures whenever informative to do so (typically when N per group is less than 10)
- For each experiment, you should identify the statistical tests used, exact values of N, definitions of center, methods of multiple test correction, and dispersion and precision measures (e.g., mean, median, SD, SEM, confidence intervals; and, for the major substantive results, a measure of effect size (e.g., Pearson's r, Cohen's d)
- Report exact p-values wherever possible alongside the summary statistics and 95% confidence intervals. These should be reported for all key questions and not only when the p-value is less than 0.05.

Please outline where this information can be found within the submission (e.g., sections or figure legends), or explain why this information doesn’t apply to your submission:

Statistical tests and models, raw data, sample sizes and p-values are reported where applicable in the text, figure captions and methods.

**Group allocation**

- Indicate how samples were allocated into experimental groups (in the case of clinical studies, please specify allocation to treatment method); if randomization was used, please also state if restricted randomization was applied
- Indicate if masking was used during group allocation, data collection and/or data analysis

Please outline where this information can be found within the submission (e.g., sections or figure legends), or explain why this information doesn’t apply to your submission:

For wild strains, individual strains are attributed to “lineages” based on an established phylogenetic definition (see figure 2a and references in the text). MA lines were attributed to “hybrid genotypes” based on the combination of haploid parental strains used to generate them.

**Additional data files (“source data”)**

- We encourage you to upload relevant additional data files, such as numerical data that are represented as a graph in a figure, or as a summary table
- Where provided, these should be in the most useful format, and they can be uploaded as “Source data” files linked to a main figure or table
- Include model definition files including the full list of parameters used
- Include code used for data analysis (e.g., R, MatLab)
- Avoid stating that data files are “available upon request”

Please indicate the figures or tables for which source data files have been provided:

Figure 2c, Figure 3a, Figure 3b, Figure 3c, Figure 4a, Figure 4b, Figure 5a-b, Figure 5c-g, Figure 6d, Figure 6f.
